# Gene expression profiles of antigenic proteins of third stage larvae of the zoonotic nematode *Anisakis pegreffii* in response to temperature conditions

**DOI:** 10.1051/parasite/2019055

**Published:** 2019-08-23

**Authors:** Marialetizia Palomba, Michela Paoletti, Alessandra Colantoni, Aurelia Rughetti, Giuseppe Nascetti, Simonetta Mattiucci

**Affiliations:** 1 Department of Public Health and Infectious Diseases, Section of Parasitology, and “Umberto I” Academic Hospital “Sapienza – University of Rome” P.le Aldo Moro, 5 00185 Rome Italy; 2 Department of Ecological and Biological Sciences, Tuscia University Viale dell’Università s/n 01100 Viterbo Italy; 3 Department of Experimental Medicine, “Sapienza-University of Rome” P.le Aldo Moro, 5 00185 Rome Italy

**Keywords:** *Anisakis pegreffii*, Gene expression, Immune-related genes, qRT-PCR, Temperature conditions

## Abstract

*Anisakis pegreffii,* a recognised etiological agent of human anisakiasis, is a parasite of homeothermic hosts at the adult stage and of ectothermic hosts at the third larval stage. Among distinct factors, temperature appears to be crucial in affecting parasite hatching, moulting and to modulate parasite-host interaction. In the present study, we investigated the gene transcripts of proteins having an antigenic role among excretory secretory products (ESPs) (i.e., a Kunitz-type trypsin inhibitor*, A.peg-1*; a glycoprotein, *A.peg-7*; and the myoglobin, *A.peg-13*) after 24 h, in *A. pegreffii* larvae maintained *in vitro,* under controlled temperature conditions. Temperatures were 37 °C and 20 °C, resembling respectively homeothermic and ectothermic hosts conditions, and 7 °C, the cold stress condition *post mortem* of the fish host. Primers of genes coding for these ESPs to be used in quantitative real-time PCR were newly designed, and qRT-PCR conditions developed. Expression profiles of the genes *A.peg-1* and *A.peg-13* were significantly up-regulated at 20 °C and 37 °C, with respect to the control (larvae kept at 2 °C for 24 h). Conversely, transcript profiles of *A.peg-7* did not significantly change among the chosen temperature conditions. In accordance with the observed transcript profiles, sodium dodecyl sulfate–polyacrylamide gel electrophoresis (SDS-PAGE) revealed the presence of the three target ESPs at 37 °C, while only *A.peg-13* was observed at 7 °C. The results suggest that temperature conditions do regulate the gene expression profiles of *A.peg-1* and *A.peg-13* in *A. pegreffii* larvae. However, regulation of the glycoprotein *A.peg-7* is likely to be related to other factors such as the host’s immune response.

## Abstract (fr)

*Anisakis pegreffii,* reconnu comme agent étiologique de l’anisakiase humaine, est un parasite d’hôtes homéothermes au stade adulte et d’hôtes ectothermes au troisième stade larvaire. Parmi les facteurs distincts, la température semble être cruciale pour l’éclosion des parasites, la mue et pour moduler l’interaction parasite-hôte. Dans la présente étude, nous avons étudié les transcrits géniques de protéines ayant un rôle antigénique parmi les produits de sécrétion excréteurs (PSE) (y compris un inhibiteur de la trypsine de type Kunitz, *A.peg-1* ; une glycoprotéine, *A.peg-7* ; et la myoglobine, *A.peg-13*) après 24 h, chez des larves de *A. pegreffii* maintenues *in vitro* dans des conditions de température contrôlées. Les températures étaient de 37 °C et 20 °C, ressemblant respectivement à la condition des hôtes homéothermes et ectothermes, et 7 °C, température de stress *post mortem* de l’hôte poisson. Des amorces de gènes codant pour ces PSE, pour utilisation dans la PCR quantitative en temps réel, ont été spécialement conçues et des conditions de qRT-PCR ont été développées. Les profils d’expression des gènes *A.peg-1* et *A.peg-13* étaient nettement régulés à la hausse à 20 °C et à 37 °C par rapport au contrôle (les larves ont été conservées à 2 °C pendant 24 h). Inversement, les profils de transcription de *A.peg-7* n’ont pas changé de manière significative parmi les conditions de température choisies. Conformément aux profils de transcription observés, la SDS-PAGE a révélé la présence des trois PSE cibles à 37 °C, alors que seul *A.peg-13* a été observé à 7 °C. Les résultats obtenus suggèrent que les conditions de température régulent le profil d’expression des gènes de *A.peg-1* et *A.peg-13* chez les larves d’*A. pegreffii*. Cependant, la régulation de la glycoprotéine *A.peg-7* est probablement liée à d’autres facteurs tels que la réponse immunitaire de l’hôte.

## Introduction

*Anisakis pegreffii* is a parasite of homeothermic hosts (mainly cetaceans) at the adult stage, while it infects ectothermic hosts (teleost fish and squids) as third stage larvae. *Anisakis pegreffii* is the dominant species of *Anisakis* in the Mediterranean Sea; in fact, it is the most common nematode in several pelagic and demersal fish of the Mediterranean Sea [43]. In Atlantic waters, the northerly limit of the *A. pegreffii* geographical range is represented by the Iberian coast. It is also widely distributed in temperate sea waters of the Austral region, between 30° S and 60° S [2, 39, 43, 59].

*Anisakis pegreffii* has been recognised as an etiological agent of human anisakiasis. Humans can acquire an accidental infection with *A. pegreffii* mainly through consumption of raw, marinated, or undercooked fish fillets (mainly anchovies in Mediterranean countries) infected by the third stage larvae of this parasite (reviewed in [25, 43]). Several cases of anisakiasis, due to the species *A. pegreffii,* have been reported in Italy [20, 22, 25, 37, 38, 41–43], Croatia [47], Japan [60] and South Korea [33]. These geographical areas, where human anisakiasis due to *A. pegreffii* has been described, correspond to areas where a high rate of parasite infection has been reported in commercial fish species [43]. The different forms of *A. pegreffii*-related disease are: gastric anisakiasis (GA), intestinal anisakiasis (IA), ectopic anisakiasis (EA), and gastro-allergic anisakiasis (GAA) (reviewed in [25, 43]).

So far, several molecular components of the L3 larvae have been identified in *A. simplex* (s. l.) as targets of human humoral IgE-mediated antigenic response (Allergen Nomenclature Sub-Committee, www.allergen.org). The antigens from *A. simplex* (s. l.) comprise both somatic antigens and excretory and secretory products (ESPs) [21, 24]. Recently, the antigens *Ani s 1 like* (24 kDa, Kunitz-type trypsin inhibitor)*, Ani s 7 like* (139 kDa, glycoprotein) and *Ani s 13 like* (37 kDa, myoglobin) were recognised in human cases of anisakiasis, as well as in IgE-hypersensitised patients due to the species *A. pegreffii* [41]. These antigens do not display amino acid sequence similarity with any other allergen able to sensitize humans. Indeed, the cross-reaction with other antigens/allergens has not been documented. Such antigens are also useful for diagnosing *Anisakis* spp. infection [24, 31, 41, 49, 58]. Other putative allergens/molecules involved in the pathogenicity have recently been suggested by an RNAseq transcriptomic analysis [5, 10, 30]. Characterisation of transcriptomes by RNAseq analysis of affected tissues from rats infected with *A. pegreffii* L3 recently indicated regulated expression modulation of host immune-related genes in response to the larval infection [8, 27, 53]. However, the expression profiles of immune-related genes in these *Anisakis* larvae in homeothermic host-infected tissue were not investigated.

Temperature has generally been accepted as one of the drivers that influences the rate of biological functions and adaptation of marine parasites [65]. Larval development and time of acclimation to an altered temperature are generally thought to be to be 5-12X slower in parasite species of ectothermic hosts living near 0–10 °C, compared with those from temperate ectothermic host species [16]. Infective larval stage of nematodes generally require specific stimuli for hatching and moulting, such as temperature, pH, and pepsin, which are known to be fundamental in the larval development to the adult stage, *in vitro* experiments [28, 48]. For many parasitic nematodes transmitted between ectothermic and homeothermic hosts – such as in the case of the *Anisakis* spp. life-cycle – the stress resulting from different host body temperatures would be a signal for further development in that host, or to maintain a homeostasis [30]. It is generally known that mRNA transcripts are a measure of signals for protein synthesis by on organism in relation to temperature [55]. Thus, the genes involved in adaptation to host temperature, parasite development, and host immune response can be differentially expressed in distinct temperature conditions. Temperature appears to modulate survival of *Anisakis* spp. [62, 63], as well as metabolic processes such as ESPs production [4]. Furthermore, temperature and storage time may play important roles in the *post mortem* motility of *A. pegreffii* larvae, indicating that storage temperature higher than 7 °C increases larval motility and therefore migration to fish muscle [14].

Recently, a comparative transcriptomic analysis of L3 and L4 *A. simplex* (s. l.) showed that several protein metabolism-related genes, likely involved in the host invading tissues, were highly expressed in L3 larvae of *A. simplex* (s. l.) [30]. In particular, the mRNA expression levels of some selected genes (i.e., *Nas-13, EF-TsMt, SFX2, dhs*) were found to be significantly higher in *A. simplex* (s. l.) third stage larvae, compared to the fourth stage larvae [30]. Furthermore, the expression patterns of Heat shock proteins (i.e., *Hsp70* and *Hsp90*) appeared to be modulated in L3 versus L4 stage larvae of *A. pegreffii,* with higher expression profiles in L4 larvae [12]. However, the expression profiles of the genes coding immuno-related proteins in L3 larvae of *Anisakis* in response to the temperature were never investigated.

The aim of this study was to investigate the impact of temperature changes on the gene expression profiles of *A. pegreffii* proteins previously found to be targets of human IgE-mediated immune response to L3 larvae, i.e. the Kunitz-type trypsin inhibitor*, A.peg-1*; the glycoprotein *A.peg-7*; and the myoglobin, *A.peg-13*. A qRT-PCR method to detect gene transcript levels was developed. Gene expression profiles were analysed in *A. pegreffii* L3, cultured *in vitro*, in response to different temperature conditions (i.e., 20 °C, 37 °C, and 7 °C), which are encountered by a third stage larva of the parasite species in ectothermic, homeothermic hosts, and under a cold stressed condition, respectively.

## Materials and methods

### Sampling and experimental design

Live *Anisakis* spp. larvae were collected from *Engraulis encrasicolus* anchovies caught in the Adriatic Sea (off the S. Benedetto del Tronto coast), where high rates of infection with the target parasite species, i.e. *A. pegreffii*, have previously been recorded [15]. After the capture, the fish were maintained at 2 °C, using a data logger, until the parasitological examination. The gentle removal of *Anisakis* spp. larvae, and checking for their integrity – the last procedure under a dissecting microscope – was performed in a refrigerated room (2 °C). Live and not disrupted larvae of *Anisakis* spp. were washed in saline solution (0.9%), several times, and then treated for 1 min, with 4% acetic acid to inhibit bacterial contamination.

In order to analyse gene expression profiles and excretory secretory products released, following temperature exposure, the larvae were cultured *in vitro* at chosen thermal conditions. These were 7 °C, temperature at which a significant increase of *A. pegreffii* motility *post mortem* of the fish host was previously observed [14]; 20 °C, average temperature of an ectothermic fish host infected by *A. pegreffii* in the Mediterranean Sea; and 37 °C, temperature of the definitive homeothermic cetacean host, as well as of the accidental human host. The gene expression and the excretory secretory products released were analysed after 24 h incubation. For each experimental condition, 50 larvae were seeded in 3.5 cm Petri Dishes in 3.5 mL of PBS 1X containing penicillin (1%) and streptomycin (1%) and incubated at the distinct temperature, 7 °C, 20 °C and 37 °C in 5% CO_2_. As control conditions, larvae (50) were also kept at 2 °C. A total of 200 *Anisakis* spp. larvae were used. The experiments were prepared in triplicate and repeated at the three temperatures.

At 24 h post-incubation, the excretory secretory products were stored at −80 °C for protein characterisation by sodium dodecyl sulfate–polyacrylamide gel electrophoresis (SDS-PAGE) and the surviving *Anisakis* spp. larvae were collected, and stored in RNA later at −80 °C for RNA and DNA extraction. A larva was considered dead if it was not motile. At 2 °C, the larval motility of *A. pegreffii* was observed to be almost absent *in vivo* [14], as well as *in vitro* experiments (Mattiucci pers. observ.).

### DNA and RNA extraction

Both DNA and RNA were extracted from each individual *Anisakis* larva used in the experiments, using TRIzol reagent (Invitrogen, Carlsbad, CA, USA), according to the manufacturer’s instructions. Briefly, each larva was homogenised using a motorised pestle and homogenised tissue in 1 mL of TRIzol, with the addition of 0.2 mL chloroform. The mixture was vortexed and centrifuged (12,000 ×*g*, 15 min, 4 °C) resulting in an aqueous (containing RNA) and organic phase (containing DNA and proteins). The aqueous layer was then transferred to a separate tube; the RNA was precipitated with 500 μL of isopropanol at room temperature for 10 min and pelleted by centrifugation (12,000 ×*g*, 15 min, 4 °C), then washed in 1 mL of 75% ethanol, centrifuged again (7500 ×*g*, 5 min a 4 °C), and dried for 10–30 min. The pellet was re-suspended in 40 μL of nuclease and RNase-free water at 60 °C for 10 min.

DNA was precipitated from the remaining interphase/organic layer with 300 μL absolute ethanol, and mixed by inverting the tube several times. The mixture was centrifuged (2000 ×*g*, 5 min, 4 °C), and the upper aqueous layer (containing proteins) was removed. The pellet was washed in 1 mL of 0.1 M sodium citrate in 10% ethanol pH 8.5, and mixed occasionally by gentle inversion. The DNA was stored in sodium citrate/ethanol for 2 h, and the mixture was then centrifuged (8000 ×*g*, 5 min, 4 °C). The supernatant was discarded with a micropipette and the pellet was re-suspended in 0.3–0.6 mL of 8 mM NaOH. The mixture was centrifuged (12,000 ×*g*, 10 min, 4 °C) and the supernatant was transferred to a fresh tube. RNA and DNA concentration and quality were measured by a NanoDrop^®^TC1-E20 spectrophotometer (BioTek Synergy HT). DNA was stored at −20 °C, while RNA was stored at −80 °C, until use.

### Molecular identification of *Anisakis* spp. larvae

Each *Anisakis* spp. larva used in the gene expression experiments was identified to species level by means of genetic/molecular markers. For this purpose, a multi-locus sequence analysis of mitochondrial (mtDNA *cox-2*) (629 bp) and nuclear (elongation factor *EF1 α−1* of nDNA) (409 bp) genes was applied.

The mitochondrial cytochrome c oxidase subunit II (*cox-2*) gene was amplified using the primers 211F (5′–TTTTCTAGTTATATAGATTGRTTYAT–3′) and 210R (5′–CACCAACTCTTAAAATTATC–3′). Polymerase chain reaction (PCR) was carried out according to the procedures previously described [39]. The sequences obtained at the mtDNA *cox-2* for those larval specimens analysed in the present study were compared with those already obtained for the same gene in the species *A*. *pegreffii* and with respect to those from other *Anisakis* spp. previously sequenced [39].

The elongation factor (*EF1 α−1* nDNA) nuclear gene was amplified using the primers EF-F (5′–TCCTCAAGCGTTGTTATCTGTT–3′) and EF-R (5′–AGTTTTGCCACTAGCGGTTCC–3′) and employing experimental conditions as previously described [40]. The sequences obtained at the *EF1 α−1* nDNA for the larval specimens analysed in the present study were compared with those already obtained for the same gene in the species *A*. *pegreffii* and *A. simplex* (s. s.), under the accession numbers KT825684 and KT825685, respectively*.* The genomic DNA obtained from those samples is stored at the Department of Public Health and Infectious Diseases (Section of Parasitology) – “Sapienza University of Rome”.

### RT-PCR, amplification of *A.peg-1*, *A.peg-7* and *A.peg-13* cDNA

Total RNA from each individual larva was treated with DNase (DNase I, Invitrogen): 1 μL DNase I (1 U/μL, Invitrogen) was added to 1 μg of RNA sample. RNA concentration and quality were measured by a NanoDrop^®^TC1-E20 spectrophotometer (BioTek Synergy HT).

High-Capacity cDNA Reverse Transcription Kits (ThermoFisher) were used for cDNA synthesis. The reverse transcription was performed in a final volume of 20 μL, in the presence of dNTP mix, (0.5 mM each), RNAse inhibitor (10 U) and with MultiScribe™ Reverse Transcriptase (4 U). Oligo (dT) and random hexamers (0.5 μM) were used as primers. The reaction was carried out in a thermal cycler at 37 °C for 2 h.

To obtain cDNA sequences of *A.peg-1, A.peg-7* and *A.peg-13* genes, species-specific primers for *A. pegreffii* larvae were first designed (Table 1), by using Primer3 web software (version 4.1.0), starting from those sequence data at the same gene coding loci, previously deposited in GenBank as *A. simplex* (s. l.) larvae [3, 24, 49]; they were synthesised by Eurofins Genomics (Ebersberg, Germany). The amplification was performed by PCR in a total volume of 25 μL containing 1× PCR buffer, 25 mM MgSO_4_, 2 mM dNTPs (each), 10 mM primer (each) (Table 1), 50 ng cDNA, 0.12 U Taq polymerase in bi-distilled water. PCR was performed as follows: 95 °C for 5 min followed by 35 cycles of 1 min at 95 °C, 1 min at different temperature of annealing for each gene (see Table 1), 1 min at 72 °C with a final extension at 72 °C for 15 min.

**Table 1:** Primer sequences of target genes coding for antigenic proteins, used in this study.

	PCR end-point			Real-time PCR		
Gene locus	Sequences (5′ – 3′)	Ta/°C	Size (bp)	Sequences (5′ – 3′)	Ta/°C	Size (bp)
*A. peg-1*	F: ATCCTCTTCACATTCGCTTT	57 °C	639	F: CATGTGCCGATAAATGCGGG	57 °C	130
	R: GGATAATAATGGTCGGGCAA			R: CCCTGTGAGCATGCATCCTT		
*A. peg-7*	F: ACACCTCCATCTGAACAAA	57 °C	899	F: TATCGGAATGCGTGACTGCA	57 °C	130
	R: CCTAACATGCAGGCGATTA			R: AGGCAGTTTCCATGGTGTATG		
*A. peg-13*	F: CATGAAATCACTCGAACACG	55 °C	931	F: AAACATTCGACGCCTACACC	60 °C	108
	R: TGTTCCTCCTTGTGCTCT			R: CATCGTGGTCTTCTCTGCGA		
*GAPDH*	–	–	–	F: CCCCTTCATCAACATCGACT	60 °C	152
				R: TCAGCTCCCCATTTGATTTC		

*GAPDH*, glyceraldehyde-3-phosphate dehydrogenase (housekeeping gene); PCR, polymerase chain reaction.

The PCR products were separated using agarose gel electrophoresis and, subsequently, the PCR products with expected molecular weight were purified, and sequenced with the same primers used for PCR (Macrogen, Europe). Nucleotide sequences of the Kunitz-type trypsin inhibitor, the glycoprotein and the myoglobin were aligned using ClustalW v2.0 [32].

### Quantitative real-time PCR

The gene-specific primers of the target genes to be used in qRT-PCR were first designed using Primer Expression 3.0 software (Applied Biosystem, USA) from the sequences of the selected *A. simplex* (s. l.) genes available in GenBank. Primer sequences of target genes used in this study in both PCR end point, and qRT-PCR are reported in Table 1. Relative gene expression profiles of L3 stage of *A. pegreffii* under different thermal conditions were evaluated by fluorescent real-time PCR. The cDNA synthesised was used as a template. The housekeeping gene glyceraldehyde-3-phosphate dehydrogenase (GAPDH) was evaluated as a reference gene; GAPDH-specific primers were employed [11].

The quantitative real-time PCR (qRT-PCR) was carried out in 50 μL of a reaction mixture with 25 μL of 2X SYBR™ Green PCR Master Mix (Applied Biosystems™) containing SYBR Green I dye, AmpliTaq Gold DNA polymerase, dUTP and buffer, 1 μL of each primer (10 Mm), 21 μL of bi-distilled water, and 2 μL of cDNA. PCR amplification was carried out using a gradient cycler StepOnePlus™ Real-Time PCR Detection System (Applied Biosystems™). The conditions used were as follows: 95 °C for 10 min, followed by 40 cycles of 95 °C for 15 s and 60 °C, according to the primers used, for 1 s. Melting curve data were collected at 55–95 °C. All reactions were performed three times, in triplicate. Amplification data were gathered and analysed. Relative expression was calculated using the ΔΔ*C*_*t*_ method [34], with all expression normalised to GAPDH levels in initial control samples. Relative levels of GAPDH were confirmed to be approximately equal across all treatments. The qRT-PCR experiment was designed and performed according to the MIQE guide recommendation [9].

### SDS-PAGE analysis

SDS-PAGE analysis was carried out on the ESPs obtained from the supernatant of cultured *A. pegreffii* larvae, collected at 7 °C, 20 °C and 37 °C, after 24 h of incubation. ESPs were concentrated as previously described [41]. Briefly, culture supernatant was mixed with 90% acetone (1:1), vortexed for 1 min, and kept in ice for 15 min. Following centrifugation at 1100 ×*g* for 10 min, the supernatant was discarded and the residual acetone was removed by evaporation at room temperature; the pellet was dissolved in PBS 1X. Protein concentration was determined using the Quick Start Bradford Protein Assay (Bio Rad), with bovine serum albumin as a standard control. For the protein preparation, samples from ESPs were individually diluted 1:1 with Laemmli buffer, and then heated at 95 °C for 5 min. Protein electrophoresis was carried out in Mini-PROTEAN 3 Cell (Bio-Rad, Hercules, CA, USA) (12% polyacrylamide separating gel with 5% stacking gel), following the manufacturer’s instructions.

### Statistical analysis

Statistical analyses were carried out using Prism8 Statistical Software from GraphPad Prism 6.0 (GraphPad Software, Inc., La Jolla, CA). Shapiro–Wilk test was performed to check the normality of the data. The one-way test was employed to compare three groups of data, while the Student’s *t-*test was used to assess the occurrence of significant differences between the two sets of data. All data were expressed as mean (*M*) ± standard deviation (*SD*). Significance was set at *p* < 0.05.

## Results

### Molecular identification of *A. pegreffii* larvae

On the basis of the sequence analysis of the mitochondrial (mtDNA *cox-2*) and nuclear (*EF1 α−1* nDNA) gene loci, the *Anisakis* L3 larvae employed in the present study were assigned to the species *A. pegreffii*. The obtained mtDNA *cox-2* sequences showed 99% or 100% similarity with sequences previously deposited in GenBank for *A. pegreffii*. Further, the same larval *Anisakis* specimens were confirmed to belong to the species *A. pegreffii*, as defined by the presence of the specific nucleotide residues present in the 409 bp length *EF1 α−1* nDNA sequences, i.e. a T and C nucleotide in position 186 and 286, respectively that have shown to be diagnostic molecular features of *A. pegreffii* [40].

### Sequencing of antigenic ESPs in L3 of *A. pegreffii*

**Figure 1:**
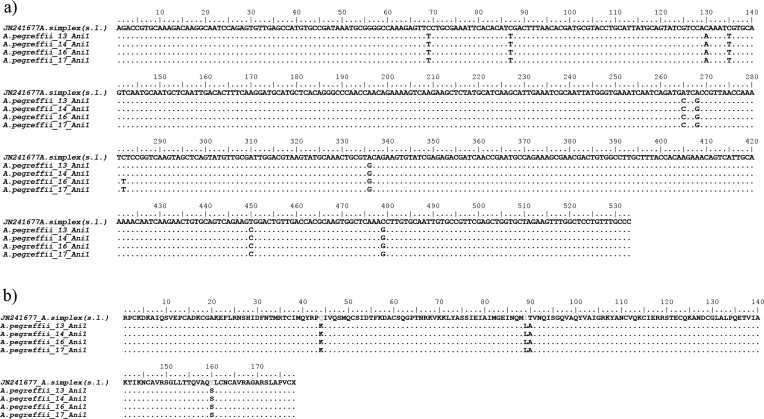
Nucleotide and amino acid sequence alignment of *A.peg-1* cDNA. (a) Nucleotide sequence alignment obtained from *A. pegreffii* larvae from the present study in comparison with those previously deposited in GenBank as *A. simplex* (s. l.) (Accession No. JN241677). Dots indicate identity with the consensus sequence. (b) Amino acid sequence alignment of the deduced amino acid sequences of the *A.peg-1* allergen showing the variable sites between *A. pegreffii*, and previously reported *A. simplex* (s. l.). Dots represent residues identical to the reference *Ani s 1* sequence.

**Figure 2:**
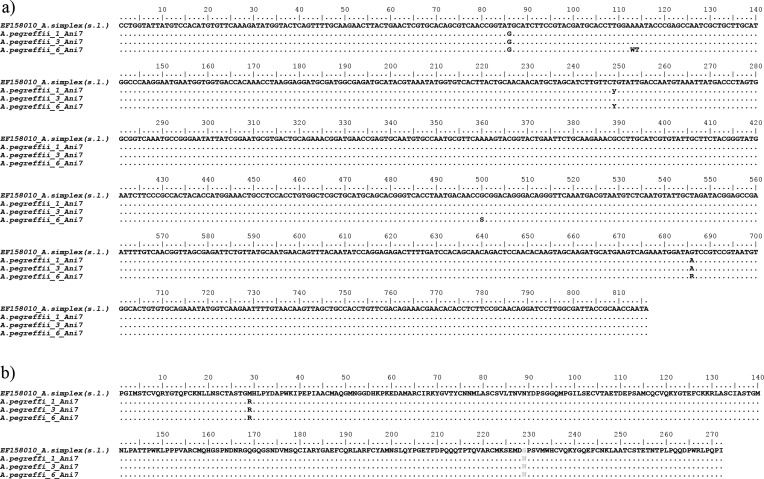
Nucleotide and amino acid sequence alignment of *A.peg-7* cDNA. (a) Nucleotide sequence alignment obtained from *A. pegreffii* larvae from the present study with respect to the internal *Ani s 7* cDNA (nucleotide [nt] 1203–2139) fragment [a potential antigenic region] of those previously deposited in GenBank as *A. simplex* (s. l.) (Accession No.: EF158010). Dots indicate identity with the consensus sequence. (b) Amino acid sequence alignment of the deduced amino acid sequences of the *A.peg-7* allergen showing the variable sites between *A. pegreffii,* and previously reported *A. simplex* (s. l.). Dots represent residues identical to the reference *Ani s 7* sequence.

**Figure 3:**
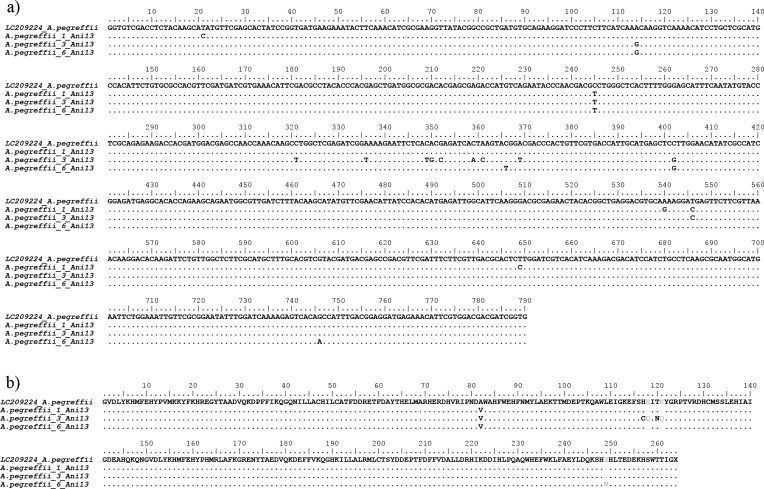
Nucleotide and amino acid sequence alignment of *A.peg-13* cDNA. (a) Nucleotide sequence alignment obtained from *A. pegreffii* larvae from the present study in comparison with those previously deposited in GenBank for *A. simplex* (s. l.) (Accession No.: LC209224). Dots indicate identity with the consensus sequence. (b) Amino acid sequence alignment of the deduced amino acid sequences of the *A.peg-13* allergen showing the variable sites between *A. pegreffii*, and previously reported *A. simplex* (s. l.). Dots represent residues identical to the reference *Ani s 13* sequence.

The *Ani s 1*, *Ani s 7* and *Ani s 13* gene sequences obtained in the present study for the species *A. pegreffii* are indicated here as *A.peg-1*, *A.peg-7* and *A.peg-13,* as previously described in the literature [1, 3, 24, 49, 54, 58]. Comparison of the sequences revealed sequences of 534 bp, 816 bp and 931 bp, respectively (Figs. 1–3). Nucleotide sequences of *A.peg-1*, *A.peg-7* and *A.peg-13* showed some nucleotide differences with respect to those previously deposited as *A. simplex* (s. l.) in GenBank. The *A.peg-1* gene locus showed 10 variable bases at positions 69, 87, 130, 135, 265, 268, 282, 336, 450 and 479, in comparison with those of *A. simplex* (s. l.) previously deposited in GenBank (Accession No. JN241677) (Fig. 1a). Four base positions 44, 89, 90 and 160 were found in the amino acid sequence alignment of *A.peg-1* in *A. pegreffii* compared to the *A. simplex* (s. l.) sequence (Fig. 1b). The *A.peg-7* gene obtained in the present study showed variable nucleotides at positions 86, 113, 114, 249, 500, and 686 in *A. pegreffii* when compared with those previously deposited in GenBank as *A. simplex* (s. l.) (Fig. 2a) (Accession No. EF158010). Two variable bases at positions 29 and 229 were found in the amino acid sequence alignment of *A.peg-7* in *A. pegreffii* with respect to *A. simplex* (s. l.) (Fig. 2b). Finally, the *A.peg-13* gene locus obtained in the present study showed several variable bp sites, with respect to that previously deposited in GenBank as *A. pegreffii* (Accession No. LC209224). Six variable bases at positions 82, 117, 118, 120, 121, and 249 were found in the amino acid sequence alignment of the protein, with respect to that available sequence (Fig. 3a and b). Sequences obtained at these gene loci for the species *A. pegreffii* were deposited in GenBank with the following accession numbers: MG962417, MG962418, MG962419, MG962420 (*A.peg-1*); MG962411, MG962412, MG962413 (*A.peg-7*) and MG962414, MG962415, MG962416 (*A.peg-13*)*.*

### Gene expression analysis

Quantitative real-time PCR analysis (qRT-PCR) revealed differential sensitivity of the three target genes in response to the exposure to the chosen temperature conditions in third larval stage of *A. pegreffii.* The Shapiro–Wilk tests supported a normal distribution of the data obtained from the qRT-PCR assay tests (all tests *p* < 0.05).

**Figure 4:**
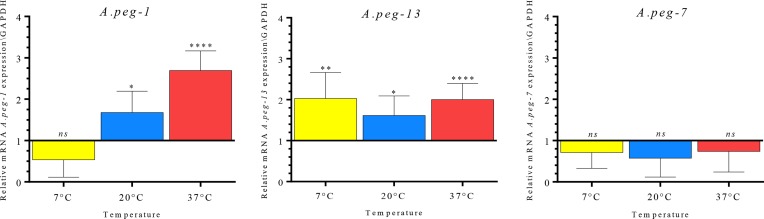
Relative expression profiles of the gene coding for the ESPs proteins, *A.peg-1, A.peg-7, A.peg-13* (shown vertically on the right side of the graph), normalised to the geometric mean of GAPDH (*gpd2*), in *A. pegreffii* larvae, in response to temperature conditions (7 °C, 20 °C and 37 °C, after 24 h). The control at 2 °C is shown as normalised to a value of 1. Each value represents the *M* ± *SD* of three biological replicates. Significant differential expression was assessed by Student’s *t* test. Significance was fixed at *p* < 0.05 [*p < 0.05, **p < 0.01, ***p < 0.001, *p* < 0.0001].

Figure 4 summarises the profile expression patterns of the *A.peg-1, A.peg-7* and *A.peg-13* genes as detected by qRT-PCR. Changes in expression levels of *A.peg-1* appeared to be highly significant (*p* < 0.0001) among the three distinct temperature exposure conditions chosen by analysis of variance (ANOVA) test (Table 2). When the comparison was performed between pair samples employing Student’s *t* test, the gene expression of *A.peg-1* after 24 h was not significantly down-regulated by exposure to a cold temperature (7 °C), as compared to the control (larvae maintained at 2 °C). However, an increase of the temperature to 20 °C and 37 °C appeared to significantly trigger the expression of *A.peg-1,* with respect to the control (*p* = 0.026, and *p* < 0.0001, respectively). Furthermore, a significant increase was observed between *A.peg-1* at 37 °C versus 20 °C (*p* < 0.001).

**Table 2:** One-way ANOVA analysis on the relative gene expression profiles of *A.peg-1*, *A.peg-7* and *A.peg-13*, in response to temperature conditions.

Gene	*df*	*MS*	*F*	*p*
*A.peg-1*	2	10.49	47.09	****
*A.peg-7*	2	0.070	0.345	*ns*
*A.peg-13*	2	0.485	1.846	*ns*

Abbreviations: ANOVA, analysis of variance; *df*, degrees of freedom; *F*, *F* ratio; *MS*, mean square; ns, not significant.

Significance was fixed at *p* < 0.05 [****p < 0.0001].

Regarding the *A.peg-13* transcripts, significantly higher gene expression was found for each temperature condition in comparison with the control. Already at 7 °C, an increase in gene expression occurred (*p* = 0.0092) and it was maintained throughout the increasing temperature, with a slight decrease at 20 °C (*p* = 0.0292), and with a maximum expression at 37 °C, when compared to the control (2 °C) (*p* = 0.0004) (Fig. 4). However, the relative gene expression of the myoglobin, did not show a significant ANOVA value (*p* = 0.1795), in comparison with the relative expression profiles reached at different temperature conditions (Table 2).

On the contrary, the transcript levels of *A.peg-7* were not significantly modulated by distinct temperature conditions (ANOVA *p* = 0.7111) (Table 2). A general trend of down-regulation of *A.peg-7* was observed at each temperature condition, as compared to the control, with a decrease, although not significant (*p* = 0.0970) (Fig. 4).

### Protein characterisation

**Figure 5:**
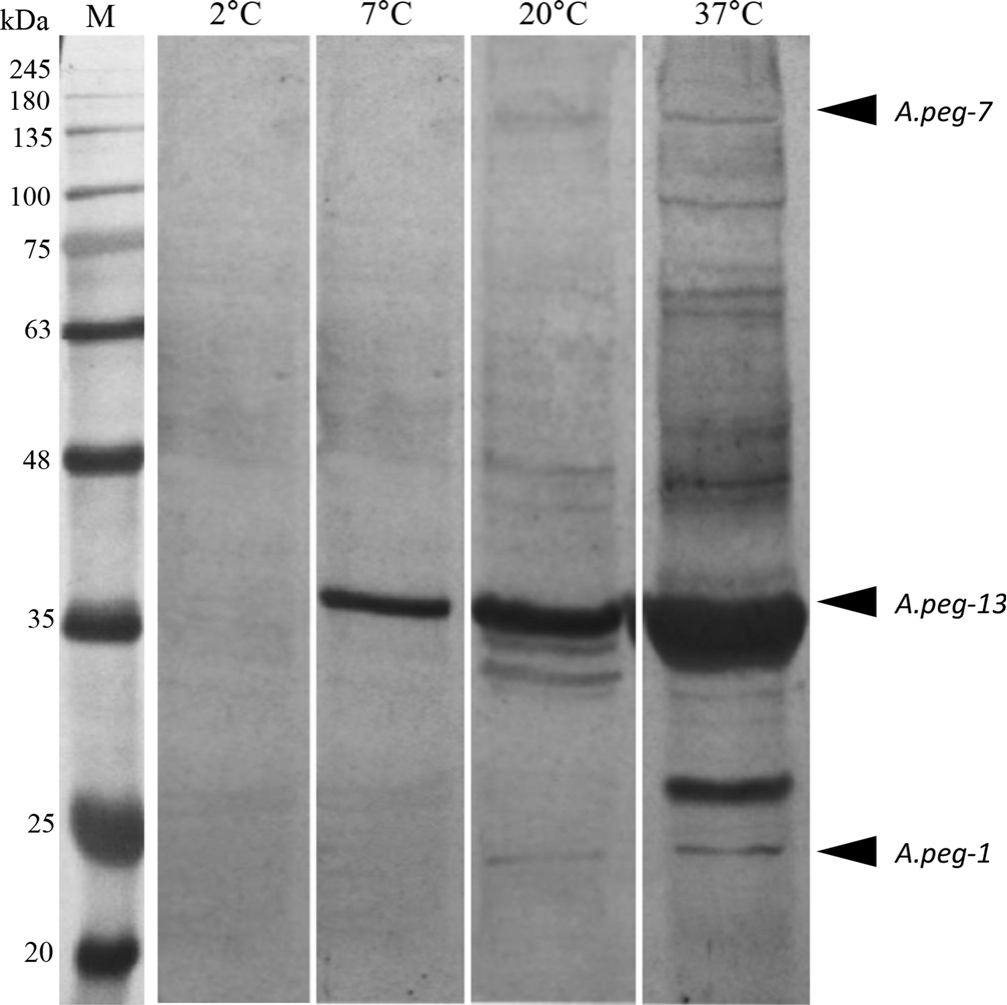
Excretory secretory proteins from advanced third stage larvae of *A. pegreffii* (*A.peg-1, A.peg-7, A.peg-13)* cultured *in vitro* in response to temperature (7 °C, 20 °C and 37 °C, after 24 h). These proteins were examined by SDS-PAGE. Supernatant of *A. pegreffii* maintained at 2 °C, used as control. M: marker.

The observed gene expression patterns were in accordance with the analysis of the relative protein levels of the parasite at the three distinct temperature conditions in SDS-PAGE. While no bands were detected in the supernatants of *A. pegreffii* larvae maintained at 2 °C for 24 h, a band likely to correspond to the *A.peg-13*, 37 kDa protein, was detected in the supernatants of larvae at the three different temperature conditions (7 °C, 20 °C and 37 °C). A 24 kDa molecule, likely corresponding to *A.peg-1*, was observed only in the culture media of *A. pegreffii* larvae maintained at 20 °C and 37 °C. Finally, another band of molecular weight around 139 kDa likely corresponding to *A.peg-7* was revealed at 37 °C, while, to a lesser extent, at 7 °C and 20 °C (Fig. 5).

## Discussion

*Anisakis* spp. larvae produce and release ESPs which may play a role during the development stages of their life cycle. In addition, the relative abundance and function of ESPs are considered to be molecular determinants in defining the host specificity of different *Anisakis* species [45]. It has also been suggested that ESPs in the third stage larvae of *Anisakis* vary quantitatively, depending on the type of physiological condition of the natural host groups (fish and mammals), as well as of the accidental ones (human host). The changing of thermal conditions experienced by *A. pegreffii* in the microhabitat of ectothermic and homeothermic hosts, involved in its life cycle, would require, among the others, molecular and biochemical responses to temperature, also as a result of ecological adaptation of the parasite species to these hosts. Indeed, for instance, temperature was found to be a sensitive parameter to trigger the development of *A. pegreffii* L3 *in vitro* cultured to L4, up to the adult stage (Mattiucci pers. observ.). A temperature-dependent relative concentration of ES proteins produced by *A. simplex* larvae, after incubation at different temperatures was previously shown [4].

The present study represents the first analysis, by using an experimental approach, on the expression patterns of genes coding for ES antigenic proteins, which were found to elicit antibody response (IgE) in humans. Indeed, target ES products in this study have already been described as the most important antigens responsible for IgE sensitisation in humans by *A. pegreffii* [41]. In *A. simplex* (s. l.), the antigen *Ani s 1* contains a Kunitz-domain [29, 35, 36, 57], with trypsin inhibitor activity and aspartic proteases [49]. Proteins with Kunitz domains are degrading enzymes that act as protease inhibitors, with a functionally conserved role in cuticle formation in a diverse range of nematodes, and possibly involved in the inhibition of thrombin and coagulation factors in the homeothermic accidental human host [44]. Proteins with Kunitz domains, showing homology with *Anis 1,* have been found in expressed transcript products obtained from pharyngeal tissues of *A. pegreffii* and *A. simplex* (s. s.) larvae [10]. These proteins have also been previously identified in studies examining the proteolytic enzymatic activity of *Anisakis* spp. [35, 36], showing an optimum of activity at the host body temperature of 36–37 °C, thus supporting the hypothesis that these enzymes are activated during the infection in homeothermic hosts of *Anisakis* spp. In accordance with these findings, we have shown that transcripts of the gene coding for *A.peg-1* from *A. pegreffii* are up-regulated in response to thermal conditions of both 20 °C, and, at a higher and significant extent, at 37 °C. While the larvae exposed to a colder thermal condition (7 °C) were still alive, the transcript levels of *A.peg-1* were observed to be down-regulated – even though not at a significant rate – with respect to the control larval samples maintained at 2 °C temperature. The up-regulation of *A.peg-1* at 20 °C and 37 °C in comparison with the control, and its release in the supernatant of *in vitro* culture, as detected by SDS-PAGE, suggests that this molecule may be related to the temperature conditions, as those found in their intermediate/paratenic fish hosts (on average, 20 °C), and in definitive, as well as in accidental human hosts (37 °C) (Figs. 4 and 5). In particular, the finding of transcripts of *A.peg-1* antigen, which was significantly released at 37 °C after 24 h, also suggests a possible role of *A.peg-1* in tissue invasion in the accidental (human) hosts. In fact, *A.peg-1* has been recognised by the IgE immune response in human serum as described in a case of gastro-allergic anisakiasis (GAA) due to *A. pegreffii* [38, 41]. Further, in an *in vivo* animal model of *Anisakis* infection, it was found that C5BL/6 mice infected with *Anisakis* spp. larvae significantly increased the production of Th17-related cytokines IL6 and IL17A, after the treatment with recombinant *Ani s 1* [13]. Another model, in which Wistar rats were exposed orally to fresh and frozen *Anisakis* larvae, showed that crude larval extracts did not induce significant IL17 by the experimentally infected host [8]. This might suggest that live larvae *A. pegreffii* and ES production of *A.peg-1* protein could trigger different immune recognition and activation pathways in accidental human hosts.

This *in vitro* study seems to indicate in addition that other molecules are up-regulated by the temperature change. The gene coding for the myoglobin *A.peg-13* is highly expressed at 37 °C and 7 °C, with respect to the control; to a lesser extent, a significant relative expression was found in the larvae exposed to 20 °C (Fig. 4). In ascaridoid nematodes, the function of *Ascaris* haemoglobin (and other nematode myoglobins) is largely unknown. It is thought that myoglobin is able to bind oxygen too tightly to be involved in its delivery, and to sequester oxygen in order to maintain an anaerobic environment [23, 46]. Furthermore, nematode myoglobin can bind and break down nitric oxide (NO) and hydrogen peroxide (H_2_O_2_), suggesting that it may provide protection against host oxidative defences [6, 23, 46]. *Anisakis* spp. myoglobin was previously found in L3 larval stages, and is associated with the excretory secretory products [53]. Also, *A.peg-13* is recognised, at high percentage, by the IgE-immune response in human cases of anisakiasis [24, 41]. The lower relative transcripts of *A.peg-13* observed at 20 °C call for future investigation of gene expression in *A. pegreffii* larvae infecting tissues of intermediate/paratenic hosts.

Differently from the other two genes in our experimental study, expression profiles of the gene coding for the glycoprotein *A.peg-7* did not change significantly at the temperatures and time intervals considered (Fig. 4, Table 2). *A.peg-7* is a glycoprotein which has been considered one of the major excretory secretory (ES) antigens, being detected in 85–100% of patients infected with *A. simplex* (s. l.) [21, 41]. Previous studies have suggested that *Anisakis* spp. larvae secrete ES products whose glycosylated components regulate the fundamental processes of antigen recognition, processing and presentation [4, 64]. On the other hand, the ES glycoprotein of the parasites can down-modulate the host immune response. For instance, in the case of *Schistosoma mansoni* eggs, an ES omega-1 glycoprotein was found to promote Th-2 skewing of dendritic cells (DCs) and T cells during infection [17]. DCs are the antigen-presenting cells (APCs) performing an essential role in the regulation and coordination of innate and adaptive immune responses. DCs are equipped with a wide range of receptors (PRRs-pathogen recognition receptors) for the recognition of parasites. Among the PRRs, the C-type lectins recognise carbohydrate structures on self and non-self glycoproteins and glycolipids [61]. In particular, macrophage C-type lectin (MGL) has been identified as a selective binder of the carbohydrate residue GalNAc-O-S/T (Tn) carried by several parasites as well as expressed by cancer cells [51, 52, 66]. Further, it has been found that the interaction between the MGL lectin expressed by dendritic cells (DCs) and the parasite *A. pegreffii* likely induces a Th-2 switch, involved in IgE-mediated responses against this parasite [50]. Thus, since the transcript levels of *A.peg-7* in *A. pegreffii* seem to be maintained in the selected temperature-conditions investigated in the present study, its gene expression would rather be modulated by the host (both of intermediate and definitive/accidental human hosts) immune response. *Ani s 7* has been suggested to be involved in the regulation of IgM response by the intermediate/paratenic host (fish) [26]. Teleost fishes are capable of producing specific immunoglobulins towards parasitic antigens, as an integrated part of adaptive immune response [7, 18, 19, 26, 56]. This would allow the larval worms to establish persistent infections in the fish host.

In our experiments, the expression profiles of this gene were down-regulated (even though not at a significant rate, with respect to the control sample), at 20 °C. On the other hand, significant differential transcripts of the gene *A.peg-7* in *A. pegreffii* larvae, from different tissues of the infected fish host, have been observed (Palomba pers. observ.), thus suggesting a possible role of the fish host immune system in the up-regulation of this gene, when infected by *A. pegreffii* larvae. Merdhana [45] demonstrated that ES products from *C*. *osculatum* activate most immune genes in a dose-dependent manner. It is noteworthy that a high concentration of *C*. *osculatum* ES proteins also induced higher expression of the fish host gene indicating activation of T regulatory cells (Tregs) which, in turn, produces inhibitory and anti-inflammatory cytokines in the host.

It has also been suggested that using the glycoprotein *A.peg-7*, the parasite *Anisakis* sp. would be able to induce and regulate the Th-2 polarising response associated with *Anisakis* infection in the accidental host (humans) [64]. On the other hand, the capacity of *A. pegreffii* larvae to impair human DC cell biology and function has also been shown experimentally [50], suggesting possible up-regulation of this gene under the effective cellular immune response of the homeothermic human host.

## Conclusions and perspectives

**Figure 6:**
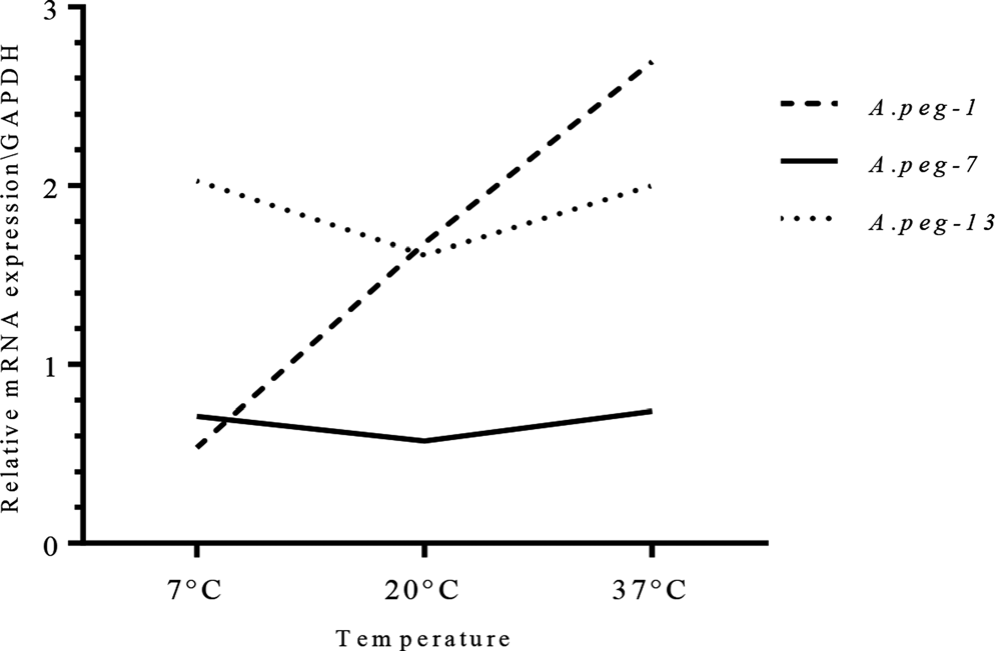
Trend of the relative expression of three selected genes coding for the ESPs proteins, *A.peg-1, A.peg-7, A.peg-13* in response to temperature conditions, using the normalisation reference gene, *gpd2*.

This study presents novel results on the expression of the *A.peg-1, A.peg-7* and *A.peg-13* genes coding for proteins among the ES products in the species *A. pegreffii*, having a major immuno-modulatory role in the host response, under the effect of temperature conditions. Particularly, a significant positive correlation between the expression levels of *A.peg-1* and *A.peg-13* was found with the temperature increase at 37 °C. This phenomenon might be able to enhance larval parasite tolerance and adaptive response to the homeothermic host microhabitat, and host immune response. On the contrary, other genes, such as the glycoprotein *A.peg-7,* were down-regulated at the chosen temperature conditions (Fig. 6).

These results seem to suggest that high temperatures may have an effect on the increase of both mRNA productions of these proteins having an adaptive role, such as *A.peg-13* and *A.peg-1* (Fig. 6)*.* Further investigation should be performed to elucidate the role of other variables, such as the influence of immunological factors of both intermediate/paratenic and accidental human hosts, as players in triggering the mRNA gene transcripts and relative protein productions, such as *A.peg-7*, as well as from other target gene-related proteins, among the ESPs produced by the zoonotic parasite *A. pegreffii*.

All three genes were not significantly expressed and released, except in the case of *A.peg-13* which is an adaptive protein, when the larvae were reared at 7 °C. The temperature range between 2 °C and 7 °C seems not to represent an optimum thermal condition for the parasite species *A. pegreffii.* In fact, as we have demonstrated in our previous studies [14], larval migration of *A. pegreffii* from the viscera to the musculature of the fish host (anchovies, *Engraulis encrasicolus*) does not occur when the fish is maintained at the temperature range comprised between 2 °C and 7 °C. Accordingly, in our experiments, the expression levels of these target genes did not significantly increase at these temperature conditions (Fig. 6). This may be important in storage of fish potentially infected with *A. pegreffii* larvae, before human consumption. The storage of fish at temperatures below 7 °C would impede not only larval motility from the viscera to the musculature, but would also induce a lower level of transcripts of genes coding for parasite-derived ES products having an immunogenic role in humans, as those considered in the present study.

Finally, quantitative RT-PCR assay of target genes would be used, in future studies, for the detection of parasite-derived gene transcripts of those antigenic proteins, in the supernatants of *in vitro* larvae maintained at 37 °C. This, in turn, would enable, in future investigations, the detection of parasite-derived secreted RNA, as possible “biomarkers” of the infection in human anisakiasis.

## Conflict of interest

The authors declare that they have no conflicts of interest.
